# Sinonasal and skull base phosphaturic mesenchymal tumours: a case series and narrative review

**DOI:** 10.3389/fendo.2026.1868955

**Published:** 2026-06-18

**Authors:** Genwang Pei, Rongfeng Lin, Guangqi Li, Yinyan Lai

**Affiliations:** 1 Department of Otorhinolaryngology–Head and Neck Surgery, The First Affiliated Hospital, Sun Yat-sen University, Guangzhou, China; 2 Department of Otorhinolaryngology, Shenzhen Baoan People’s Hospital, Shenzhen, China

**Keywords:** hypophosphataemia, misdiagnosis/missed diagnosis, phosphaturic mesenchymal tumour, sinonasal, skull base

## Abstract

Phosphaturic mesenchymal tumours (PMTs), which are common causes of tumour-induced osteomalacia (TIO), are exceptionally uncommon in the sinonasal tract and skull base. PMTs are characterized by their nonspecific location, small size, and indolent growth. When they occur in the sinonasal region and skull base, they often lack typical sinonasal symptoms, which poses significant diagnostic challenges and contributes to high rates of missed and misdiagnosis. A retrospective analysis was conducted on 5 patients with pathologically confirmed sinonasal/skull base PMTs treated at our institution from December 2015 to March 2025. The study cohort comprised three male and two female patients aged 28 to 67 years. The sites of involvement were the left sinonasal cavity (n = 3), right sinonasal cavity (n = 1), and sinonasal skull base (n = 1), with all patients undergoing surgical resection of the lesions. Our findings indicate that the increased incidence of missed and misdiagnoses primarily arises from tumour obscurity, atypical clinical manifestations, and insufficient clinical recognition of the condition. Persistent hypophosphataemia provides vital diagnostic information. PET demonstrating somatostatin receptor positivity and immunohistochemistry revealing SSTR2+/SATB2+ expression are key auxiliary diagnostic tools. Monitoring postoperative serum phosphorus levels can aid in the effective evaluation of cure and recurrence.

## Introduction

Phosphaturic mesenchymal tumour (PMT) is an important aetiological factor in tumour-induced osteomalacia (TIO). The underlying pathogenesis involves tumour cell secretion of fibroblast growth factor 23 (FGF23), which targets renal tubules to inhibit phosphate reabsorption. These alterations induce hypophosphataemia with hyperphosphaturia, ultimately impairing bone mineral homeostasis. Clinically, this manifests as osteomalacia characterized by diffuse musculoskeletal pain, proximal muscle weakness, and fragility fractures. PMTs most commonly occur in soft tissues and bones, particularly in the extremities, with sinonasal and skull base locations being relatively rare. Owing to their slow growth, small size, and anatomically obscured location within the sinonasal cavity and skull base, these tumours are prone to missed or misdiagnosis. Furthermore, the complex regional anatomy, involving critical neurovascular structures and proximity to the skull base, renders surgical intervention highly challenging. This article presents the diagnosis and management of five cases of sinonasal/skull base PMTs from our institution. It aims to summarize clinical experience, identify critical diagnostic indicators for this condition, and analyse current challenges and potential improvements. By reflecting on instances of missed and misdiagnosis, this study seeks to optimize the diagnosis and management of this rare disease.

Cases report: See ‘Baseline Data of 5 Patients’ table for details (**Table 1**).

**Table 1:** Baseline data of 5 patients.

Baseline data of 5 patients	Gender	Age	Present illness	Nasal symptoms	Past medical history	Laboratory data	Tumor location	treatment option	Recovery time of phosphatemia	Pathology	Imaging findings	Detection method	Initial department	Nasal endoscopy
1	female	43	Weakness in bilateral lower extremities for 6 months	no	Bone pain, rib and femoral fracture history	PHOS 0.54↓ mmol/L 1,25(OH)₂-Vitamin D, iPTH, urinary phosphate, BALP, and serum calcium within normal ranges.	Left ethmoid sinus	Surgery	postoperative day 4	short spindle cells, CD34(-), Actin (rare cells +), S-100(-), EMA (focal weak +), Desmin(-), STAT-6(-), SSTR2 (partial +), SATB2(+)	MRI: Oval mass, T1: iso-/mildly hypointense. T2: mildly hypointense. Heterogeneous enhancement. Bone destruction. CT: Oval mass, Adjacent bone compression and destruction. 68Ga-DOTANOC PET-CT: Left ethmoid sinus soft tissue nodule, positive somatostatin receptor imaging, no bone destruction.	PET-CT	endocrinology	nasal cavity mass
2	male	41	Bone pain for 6 months	no	Bone pain, rib fracture history	PHOS 0.70 ↓ mmol/L。 1,25(OH)₂D 18 ↓ ng/mL BALP 26.69 ↑ μg/L Urinary phosphate and serum calcium within normal ranges.	Left ethmoid sinus	Surgery	postoperative day 5	short spindle cells,Actin (partial +), β-Catenin(-), CK(-), Vimentin(+), EMA(-), SSTR2 (partial +), Syn(-), CgA(-), S-100 (rare cells +), Ki-67 (approximately 5%+), CD34(-), Bcl-2(+)	MRI: Oval mass, T1: isointense, T2 :mildly hyperintense. Marked enhancement. No bone destruction. CT: Oval mass,Marked enhancement, No bone destruction. PET-CT: positive somatostatin receptor imaging.	PET-CT	nuclear medicine	normal
3	female	67	Left nasal and skull base mass for 1 month	Nasal obstruction ,intermittent epistaxis	Bone pain, rib fracture history	1,25(OH)₂D 18↓ ng/mL PHOS 0.76↓ mmol/L iPTH, serum calcium, and calcitonin within normal ranges.	Skull base	Surgery	postoperative day 7	short spindle cells,Low-grade malignant,SSTR2(+),SATB2(+),CD34(-),Actin(-),S-100(-),EMA(-),Myogenin(-),MyoD1(-),CK(-),HMB-45(-)。	CT: anterior perpendicular plate bone destruction with a hypervascular soft tissue mass, invading bilateral frontal sinuses and intracranial cavity, an aggressive neoplastic lesion, Heterogeneous enhancement.	nasal endoscopy	otolaryngology	nasal cavity mass
4	male	28	Left nasal cavity mass for 1 month	Nasal obstruction	Bone pain with multiple fractures involving ribs, femurs, fibulae, ilium, and other bones.	PHOS 0.48↓mmol/L,1,25(OH)₂D 21↓ ng/mL BALP 106.50↑ μg/L iPTH, urinary phosphate, and serum calcium within normal ranges.	Left ethmoid sinus	Surgery	postoperative day 5	short spindle cells,CD34(-), STAT-6(-), CK(-), B-Catenin(-), PR(-), Ki-67 (approximately 10%+), Actin (partial +), Desmin(-), MyoD1(-), Myogenin(-), S-100(-), SOX10(-), SSTR2(+), ERG (focal weak +)	CT: Oval mass, Heterogeneous enhancement. MRI: T1: iso-/mildly hyperintense. T2 and fat-suppressed T2: hyperintense.Marked enhancement; No bone destruction. 18F-FDG PET-MRI: soft tissue nodule in left ethmoid sinus-nasal passage, increased glucose metabolism, positive somatostatin receptor imaging;	PET-MRI	endocrinology	nasal cavity mass
5	male	43	Right nasal cavity mass for 2 weeks	Nasal obstruction	Osteoporosis with generalized pain for over 1 year and history of rib fractures	PHOS 0.54↓ mmol/L,1,25(OH)₂D 21↓ ng/mL BALP 36.78↑ μg/L Urinary phosphate, iPTH, and serum calcium within normal ranges.	Right ethmoid sinus	Surgery	postoperative day 4	short spindle cells,Vimentin(+), SSTR2(+), EMA (focal weak +), CD56(+), CD99(+), CK(-), CD34(-), S-100(-), STAT-6(-), Actin/SMA(-), β-Catenin(-), Desmin(-), Syn(-), CgA(-), Ki-67 (approximately 5%+)	CT: Bone resorption and destruction. MRI: T1: isointense, T2 :mildly hyperintense , relatively homogeneous signal; Internal linear hypointense shadows. Marked and relatively homogeneous enhancement 18F-FDG PET-CT: positive somatostatin receptor imaging.	PET-CT	endocrinology	nasal cavity mass

BALP, Bone Alkaline Phosphatase; iPTH, Intact Parathyroid Hormone. ↑ stands for an increase relative to the normal value, while ↓ stands for a decrease relative to the normal value.

From December 2015 to March 2025, five cases of sinonasal/skull base PMTs were diagnosed at our institution. The cohort comprised three males and two females. The tumour distribution revealed one skull base lesion and four ethmoid sinus tumours—three in the left ethmoid sinus and one in the right. All patients (5/5) presented with a history of bone pain and pathological fractures, predominantly involving the ribs. One patient sustained multifocal fractures (ribs, ilium, femur, and fibula) (Case 4 in the ‘Baseline Data of 5 Patients’ table). The mean diagnostic delay from symptom onset to tumour identification was several years. A review of the cases revealed that among the 5 patients, 4 had specific durations from symptom onset to diagnosis, ranging from 0.5 to 5 years, with an average of 3 years. One case lacks specific details and is described only as “several years”. Nasal symptoms—including obstruction and intermittent epistaxis—were reported in three patients. Nasal endoscopy revealed masses in four patients. All patients underwent endoscopic surgery (5/5). One patient experienced recurrence after 2 years and underwent reoperation. Serum phosphorus levels normalized within 4–10 days post-operatively in all patients.

Laboratory data: Serum phosphorus concentration (5/5 decreased, 0.48–0.76 mmol/L; reference range: 0.97–1.62 mmol/L), 1,25-hydroxyvitamin D concentration (4/4 decreased, 18–21 ng/mL; 1 case not tested; reference range: >25 ng/ml), bone alkaline phosphatase (BALP) concentration (3/4 elevated, 26.69–106.50 µg/L; 1/4 normal, 1 case not tested; reference range: 3.87–20.73 µg/L), urine phosphorus concentration (4/4 normal, 1 case not tested; reference range: 3.4–38.9 mmol/L), intact parathyroid hormone (iPTH) concentration and serum calcium concentration (5/5 normal).

**Figure 1:**
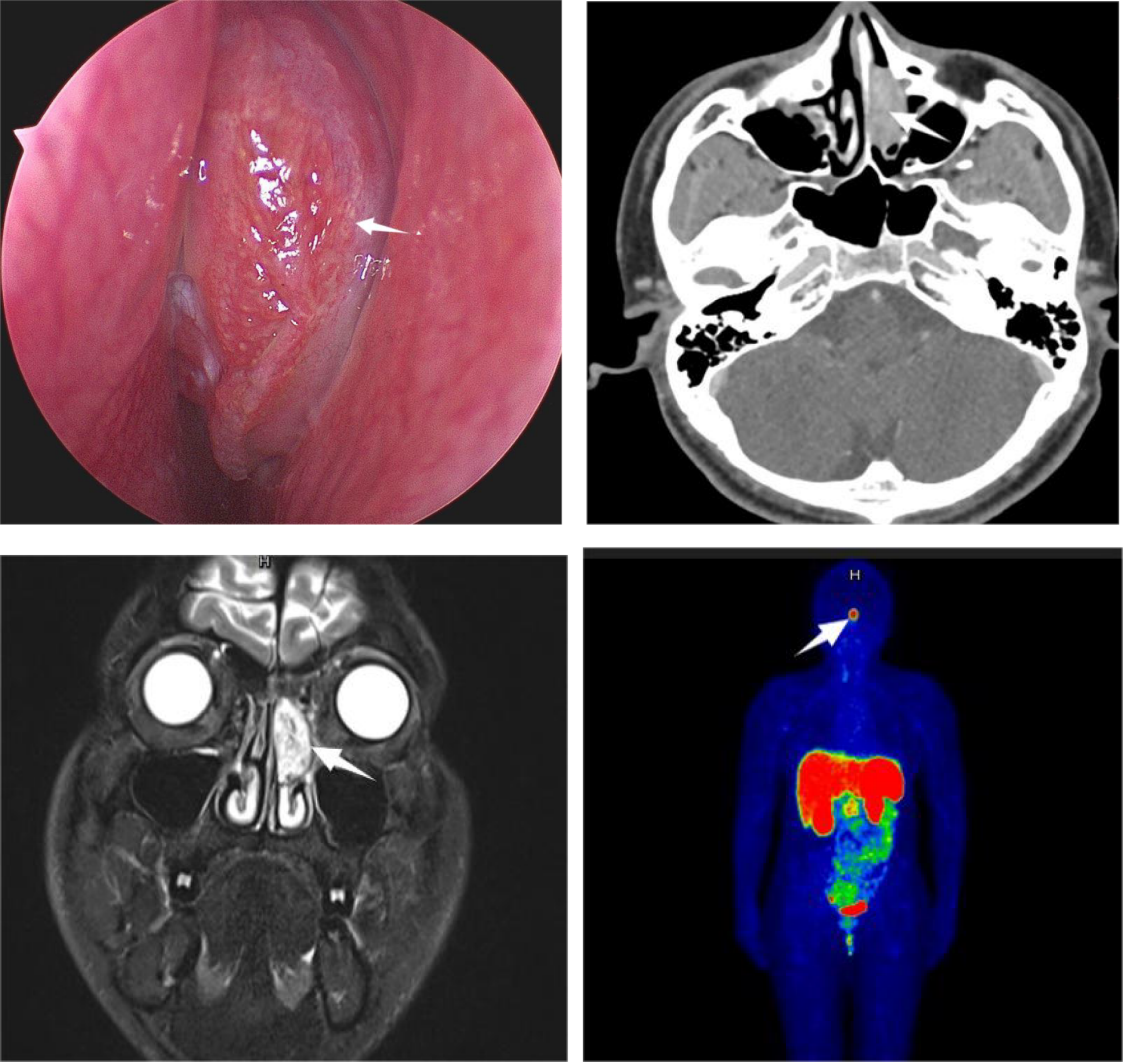
Typical imaging findings (case 4) (Endoscopy/CT/MRI/PET, white arrow: tumour).

Imaging data: CT: Preoperative CT was performed in all 5 patients, while MRI and PET were conducted in 4 patients. Imaging findings suggested bone destruction in 3 patients. Benign lesions predominantly presented as oval-shaped lesions with heterogeneous enhancement, suggesting hypervascular tumours; adjacent bone demonstrated compressive remodelling. Malignant lesions exhibit invasive bony destruction. MRI: T1-weighted imaging revealed a hypointense to isointense signal; T2-weighted imaging predominantly demonstrated a hyperintense signal. Heterogeneous enhancement was observed in all the patients. PET revealed positive somatostatin receptor expression (**Figure 1**).

**Figure 2:**
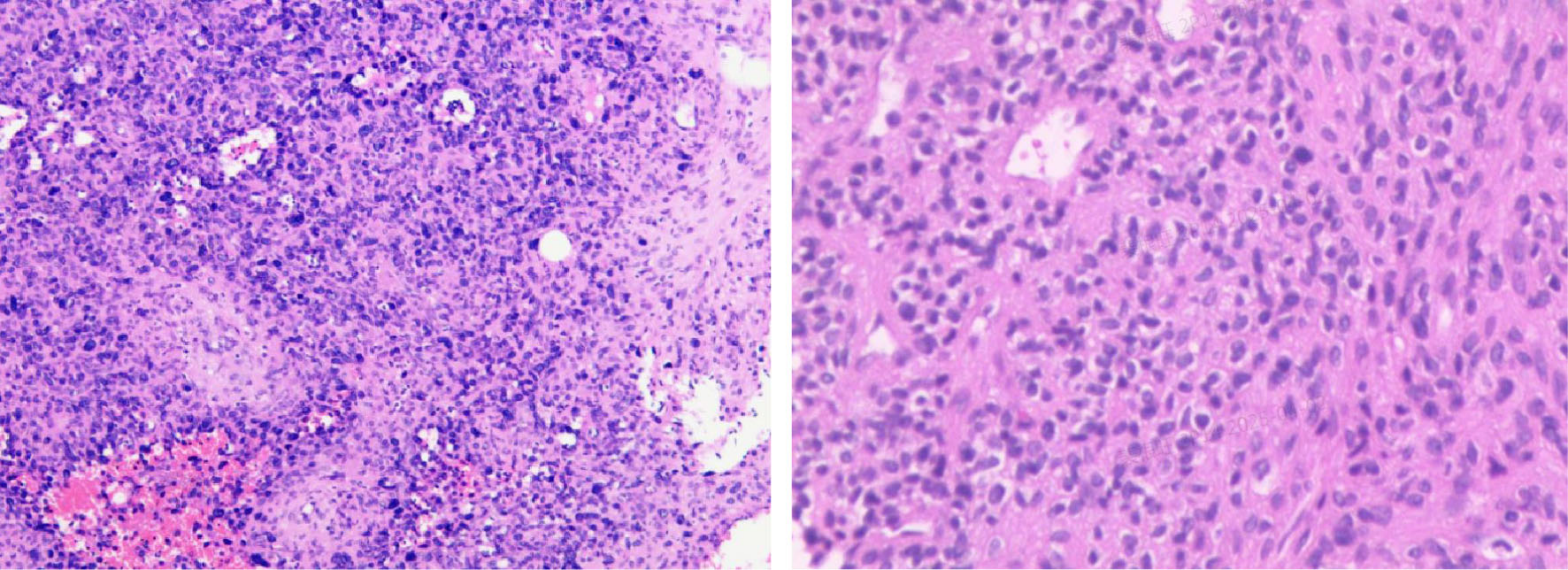
Typical pathological features (case 4) (the cells are short, spindle shaped and densely arranged).

Pathology: Four cases were benign, and one was low-grade malignant (Case 3 in the ‘Baseline Data of 5 Patients’ table). Both benign and malignant diagnoses were verified by pathological tissue examination. Histopathological examination revealed that the tumour was predominantly composed of short spindle cells, with a stroma rich in vascular networks. Focal areas exhibit a haemangiopericytoma-like pattern, and calcification is observed. The immunohistochemistry results were as follows: SSTR2 positive or partially positive (5/5), SATB2 positive (2/2), actin focally positive (3/5), EMA weakly positive (2/4), vimentin positive (1/1), CD56 positive (1/1), and Ki-67 approximately 5%-10% (3/3). CD34, S-100, EMA, desmin, STAT-6, β-catenin, CK, Syn, CgA, myogenin, MyoD1, HMB-45, and SOX10 were all negative. The pathology for Case 4 is shown in **Figure 2**.

Other data: Five patients were predominantly diagnosed through PET imaging (4/5). Initial clinical evaluation occurred in the Endocrinology (3/5) and Nuclear Medicine (1/5) departments; only a single case was incidentally detected via nasal endoscopy in Otorhinolaryngology. All the patients (5/5) exhibited prior diagnostic delays, including missed or misdiagnosed presentations. Misdiagnoses included nasal polyps and nasal haemangioma, which were particularly typical in the recurrent case (Case 5 in the ‘Baseline Data of 5 Patients’ table). The patient initially presented to an external hospital; sinus CT suggested nasal polyps, and no surgery was performed. One year later, surgery was performed because of nasal obstruction, and postoperative pathology suggested nasal angiofibroma. Five years later, the patient first visited our hospital, and a pathology slide consultation revealed a diagnosis of PMT. The patient underwent tumour resection and was placed on a regimen of periodic follow-up. Recurrence of hypophosphataemia was noted two years post-operatively, prompting an MRI that confirmed tumour recurrence.

## Discussion

Tumour-induced osteomalacia (TIO) was initially described in 1985. These mesenchymal-origin tumours universally induce phosphaturia and hypophosphataemia, leading to their designation as phosphaturic mesenchymal tumours (PMTs). The primary pathogenic mechanism involves tumour-derived fibroblast growth factor 23 (FGF23), which inhibits renal tubular phosphate reabsorption. This results in persistent hypophosphataemia, typically accompanied by phosphaturia, ultimately disrupting bone mineralization. Concomitant suppression of 1,25-dihydroxyvitamin D [1,25(OH)_2_D] synthesis impairs osteoid mineralization, which manifests clinically as osteomalacia with musculoskeletal pain, proximal muscle weakness, and pathological fractures (1). Lesions most commonly occur in the long bones of the limbs, with head and neck involvement accounting for less than 5% of the lesions (2). Within the head and neck region, the sinonasal cavities and the skull base represent the predominant sites for PMTs. Tumours arising in these locations are typically small and anatomically obscured. Moreover, patients often lack sinonasal symptoms, leading to initial presentation in primary care settings—frequently to orthopaedic departments for musculoskeletal complaints. Sinonasal/skull base PMTs are frequently detected incidentally during systemic PET evaluations or only upon manifestation of local symptoms such as nasal obstruction or epistaxis. Given the indolent growth pattern of tumours, the clinical course often extends over years. Throughout this prolonged diagnostic process, patients endure persistent morbidity related to osteomalacia, including recurrent pathological fractures. The time from symptom onset to definitive diagnosis is often reported to exceed 5 years (3). Typical presentations of sinonasal PMTs include systemic symptoms such as musculoskeletal pain, weakness, and multiple fractures, as well as local symptoms such as epistaxis and nasal obstruction. Characteristic laboratory findings typically include low serum phosphorus levels (usually with normal random urine phosphate levels), low 1,25-hydroxyvitamin D levels, normal serum calcium levels, and normal iPTH levels. Since serum phosphorus and calcium are commonly included in routine biochemical screening, identifying the underlying cause of hypophosphataemia is crucial. Normal random urine phosphate levels were observed. This finding can be explained by the inherent limitations of random urine testing compared with the gold-standard 24-hour collection, which we anticipate would be abnormal. Furthermore, it may reflect the typically small volume and consequently limited hormone secretory capacity of sinonasal skull base tumours or the activation of an unknown systemic compensatory mechanism. Therefore, we recommend 24-hour urine phosphate monitoring and further investigation into the underlying pathophysiology.

Surgical resection is the primary treatment for tumours in this location. Serum phosphorus levels typically normalize within approximately one week post-operatively; in our study, all five cases normalized within 10 days following surgery, with other symptoms resolving over time. All five patients in our study achieved favourable surgical outcomes. In the recurrent case, serum phosphorus levels normalized 4 days after the initial resection at our institution and 10 days after the subsequent resection. This phenomenon might have occurred because surgical removal of the majority of the tumour tissue significantly compromised its FGF-23 secretion capacity. The patient was followed up for 2 years with sustained normal phosphorus levels and no evidence of recurrence. We propose that regular postoperative monitoring of serum phosphorus could serve as a tool to assess tumour recurrence.

Our findings indicate that sinonasal tumours most commonly originate in the ethmoid sinus, which aligns with the findings of Kane et al., who reported the ethmoid sinus as the origin in 64.7% of cases (4). Owing to the complex sinus anatomy, precise localization to a single anatomical site may pose challenges. Histologically, the tumors were composed predominantly of short spindle-shaped cells. Immunohistochemically, all cases expressed SSTR2, and SATB2 was positive in the two cases tested. These findings are consistent with those reported by Agaimy (5)who demonstrated uniform expression of SSTR2A, ERG, and CD56 in the vast majority of PMTs. The Ki-67 index was consistently low (5–10% in the three cases evaluated), which is likely attributable to the predominantly benign or low-grade malignant nature of the tumors in our series. One of our cases tested positive for CD56 (1/1), further supporting the immunophenotypic profile of PMT, which frequently co-expresses SSTR2, SATB2, and CD56 (6). However, Agaimy’s cohort included predominantly extremity and soft tissue PMTs, whereas our series is restricted to sinonasal and skull base tumors. Whether the sinonasal subset exhibits an identical immunohistochemical profile remains to be determined. Nevertheless, given the limited size of our cohort, these observations require validation in larger, preferably multi-institutional studies.Lee JC and colleagues identified FN1-FGFR1 gene fusion in PMT tumour specimens via RNA sequencing (7). On the basis of the imaging findings, we observed that CT and MRI lack distinctive characteristic changes, demonstrating heterogeneous signal presentations. However, positive somatostatin receptor imaging on PET represents a characteristic finding and can function as an important auxiliary tool for definitive diagnosis (8). Serum phosphorus levels can be used as a tumour surveillance indicator, particularly for postoperative recurrence. Patients who experience persistently nonnormalized serum phosphorus levels postoperatively merit further investigation. Synthesizing our observations, we propose the following key diagnostic markers for PMT: persistent hypophosphataemia + characteristic pathological immunohistochemistry (SSTR2+) + characteristic PET somatostatin receptor positivity.

All 5 cases in our study were missed or misdiagnosed. A review of domestic and international clinical data suggested a missed/misdiagnosis rate exceeding 95% (9). Considering that all the reported cases were ultimately diagnosed in tertiary hospitals, and summarizing our own cases where most were discovered via PET, we believe that the high rate of missed/misdiagnosis may be related to the diagnostic capabilities and basic hardware of local hospitals, as well as a lack of systematic training on rare diseases. It is necessary to strengthen the relevant training.

## Limitations

Several limitations of this study should be acknowledged. First, this is a retrospective case series with a small sample size (n=5) from a single tertiary referral centre, which may introduce selection bias and limit the generalizability of our findings. Second, serum fibroblast growth factor 23 (FGF23) levels were not measured in any of the five cases because of the retrospective design spanning from 2015 to 2025 and the unavailability of stored blood samples for referred patients. Although our diagnoses were supported by consistent clinical, biochemical, imaging, and histopathological criteria—including rapid postoperative normalization of serum phosphorus—the lack of FGF23 confirmation remains a notable gap. Third, random urine phosphate was used instead of the gold−standard 24−hour urine collection, which might have led to the underestimation of phosphaturia. Fourth, the long diagnostic delays and frequent misdiagnoses observed in our series may partly reflect referral bias to a tertiary hospital. Finally, as a descriptive case series without a control group or prospective follow−up protocol, causal inferences regarding diagnostic markers or surgical outcomes should be drawn with caution. Future prospective studies with larger cohorts, routine FGF23 measurements, and standardized long−term follow−up are warranted to validate our observations.

## Data Availability

The original contributions presented in the study are included in the article/supplementary material, further inquiries can be directed to the corresponding author/s.
